# Subsequent Fertility of Goats with Prenatal Mortality Diagnosed by Ultrasound and Treated by PGF_2*α*_ and Oxytetracycline

**DOI:** 10.1155/2017/7890183

**Published:** 2017-01-02

**Authors:** A. S. Aban, R. M. Abdelghafar, M. E. Badawi, A. M. Almubarak

**Affiliations:** ^1^Department of Surgery and Gynecology, Faculty of Veterinary Medicine, Upper Nile University, Malakal, South Sudan; ^2^Department of Veterinary Medicine and Surgery, College of Veterinary Medicine, Sudan University of Science and Technology, Khartoum, Sudan

## Abstract

Thirteen Saanen and Saanen crossbred female goats, between the ages of 6 months and 7, years were presented to the clinic, College of Veterinary Medicine, Sudan University of Science and Technology, for sonographic pregnancy diagnosis. Transabdominal ultrasound was performed using 3.5 MHz probe which revealed non-viable fetuses as judged by absence of heart beats and movements. Twelve goats were given single i/m injection of PGF_2*α*_ analogue and 5% oxytetracycline. Ten goats responded to the treatment and six of them became pregnant and gave birth within the normal gestational period. One goat was diagnosed as non-pregnant, one goat developed hydrometra, and the subsequent fertility of two goats was unknown. Two full-term goats did not respond to treatment. Another dose of PGF_2*α*_ was administered to them and again they did not respond. Manual attempts were done to deliver the full-term goat with dilated cervix and they were unsuccessful. Cesarean section and hysterectomy were then performed for the three full-term goats with unfavorable outcome. It can be concluded that ultrasound is a rapid, reliable, and nonhazardous procedure for the diagnosis of fetal mortality in goats and PGF_2*α*_ treatment in conjunction with oxytetracycline is an efficient treatment.

## 1. Introduction

Sudan possesses about 31 million goats [1]. Small ruminants, especially goats, are very important in rural economy and have potentially been used as a tool for poverty alleviation [2]. Fetal death is defined as cessation of heart beats and absence of fetal movement [3, 4]. Embryonic and fetal mortality contribute to large economic loss [5]. Economic losses resulting from fetal death are substantial since they include not only the loss of offspring but also a prolonged open period for the dam leading to increased culling rates [4]. The high rate of embryonic loss (25–40%) in domestic species during early pregnancy may result in false positive diagnosis [6]. The causes of fetal death are multifactorial and can be divided broadly into infectious and non-infectious origin with the most frequently detected infectious agents being bacteria, viruses, fungi, and parasites. Non-infectious causes of fetal death include malnutrition, stress, maternal endocrine imbalance, and ambient temperature [7–9]. Several infectious agents that cause fetal death and abortion are zoonotic, for example,* Brucella*,* Listeria*,* Coxiella*,* Chlamydia*, and* Toxoplasma* [7].

Early and accurate diagnosis of pregnancy, determination of litter size, and estimation of gestational age in livestock are crucial for improving efficiency of reproduction in goats [10–13]. Ultrasound technique has become an essential tool in veterinary medicine for the evaluation of intrauterine life of the fetus [14]. Lack of echogenicity of amniotic fluid, the proper amount of fluid for the gestational stage, and normal fetal posture and movement are signs of a healthy fetus [15].

Fetal size incompatible with the expected gestational age may indicate earlier fetal death. Absence of heart beats and movements, increased fluid echogenicity, collapsed fetal posture, and hyperechogenicity of the cotyledons are a common finding in a non-viable pregnancy [7, 15]. If fetal degeneration occurs, sonographic image will demonstrate an undifferentiated image of the uterine content with anechoic to hyperechoic structure [16]. The exact outcome of antenatal death is unpredictable and is influenced by several factors, including the cause of fetal mortality, differences in pregnancy between species, stage of gestation at fetal death, and number of fetuses [17]. Early embryonic death with the loss of corpus luteum produces a subsequent return to estrus following resorption of the embryonic material [15]. However, the outcome in case of failure of the corpus luteum to undergo luteolysis may be prolonged gestation, pyometra, or fetal mummification [18, 19]. In cases of abortion and fetal maceration, the hormonal support of pregnancy is lost [20]. Major infectious agents of abortion in goats are* Chlamydia*,* Toxoplasma*,* Leptospira*,* Brucella*,* Coxiella burnetii*, and* Listeria*. Non-infectious causes of abortion may be genetic, chromosomal, hormonal, and nutritional. Nutritional factors include plant toxins, such as broom weed or locoweed poisoning; dietary deficiencies of copper, selenium, vitamin A, and magnesium; certain drugs such as estrogen, glucocorticoids, phenothiazine, carbon tetrachloride, and levamisole in late gestation [3, 20].

Prostaglandins are secreted by almost all body tissues. PGF_2*α*_ is the natural luteolytic agent that terminates the luteal phase of the oestrous cycle and allows for the initiation of a new one in the absence of fertilization, and it is particularly potent in terminating early pregnancy [3].

Administration of prostaglandin F_2*α*_ and its agonist cloprostenol is an efficient treatment of fetal death in does [21]. It has been concerned in the changes that occur in the connective tissue of the cervix at labor onset and stimulates contractions of the uterus [3, 22]. Unlike other ruminants where placenta-derived progesterone becomes significant, the goat depends on corpus-luteum-derived progesterone throughout pregnancy and is thus susceptible to luteolytic agents, including prostaglandins, throughout the whole period of pregnancy [15].

To the best of the authors' knowledge, few case reports have been published in the Sudan regarding diagnosis of fetal death in goats using ultrasound technique [23, 24]; however, treatment, follow-up, and subsequent reproductive performance were not reported. Thus, the aim of the current research was to report sonographic diagnosis of antenatal mortality, treatment, and consequent reproductive performance in goats for the first time.

## 2. Materials and Methods

### 2.1. Animals

Two nulliparous and 11 pluriparous (*n* = 13) Saanen and Saanen crossbred female goats were included in the present study. Their ages were between 6 months and 7 years. The goats were presented to the Veterinary Teaching Hospital, College of Veterinary Medicine, Sudan University of Science and Technology (SUST), during the period of March 2015-2016 for sonographic pregnancy diagnosis. Ten goats presented for routine pregnancy diagnosis because of absence of estrus and three goats were presented because they did not give birth at the estimated gestational age as determined early by ultrasonography.

### 2.2. Methods

#### 2.2.1. Clinical Examination

Full clinical examination was done for all goats. Ten goats were alert, in a good condition and all physiological parameters were within the normal range. Regarding the three full-term goats, one of them had a hard palpable immobile abdominal mass, the os cervix was closed, respiratory and heart beats were normal, and the general condition of the goat was not altered. The second goat had dilated cervix and fetid bloody stained vaginal discharge was realized. The general condition of the goat was poor with greatly increased heart beats and respiratory rate. The 3rd goat had a closed cervix.

### 2.3. Ultrasound Scanning

#### 2.3.1. Animal Preparations

Animals were deprived from food for 12 hours prior to the scanning to avoid accumulation of gases into the gastrointestinal tract. Area of scanning which extends across the width of the abdomen, passing from one side of the udder, a cross the abdomen in front of the udder, to the other side and 15 cm anterior to the udder [25] was clipped and shaved carefully using manual clippers (Super-Max, Green, Feltham, London TW13 7LR, UK). A copious amount of ultrasonic gel (Aquasonic, Parker Laboratories, Inc., Fairfield, NJ 07004, USA) was applied to the ventral abdomen prior to scanning.

#### 2.3.2. Animal Positioning

Animals were turned on their backs (dorsal decubitus) and well restrained on especially designed table.

#### 2.3.3. Machine and Image Recording

Transabdominal ultrasonography was performed using a real-time ultrasound scanner (Pie Medical, Esaote, Netherlands) equipped with dual frequency (3.5–5) MHz convex transducer. Sagittal, parasagittal, and cross sections were taken to ascertain accurate diagnosis. Images were stored in a memory card attached to the scanner and later were printed in thermal papers (Sony Corporation, type 1, Normal, UPP-110S, 1-7-1, Konan, Minato-ku, Tokyo, Japan) using video graphic printer UP-895EC (Sony, Japan).

## 3. Results

### 3.1. Ultrasound Scanning

**Figure 1:**
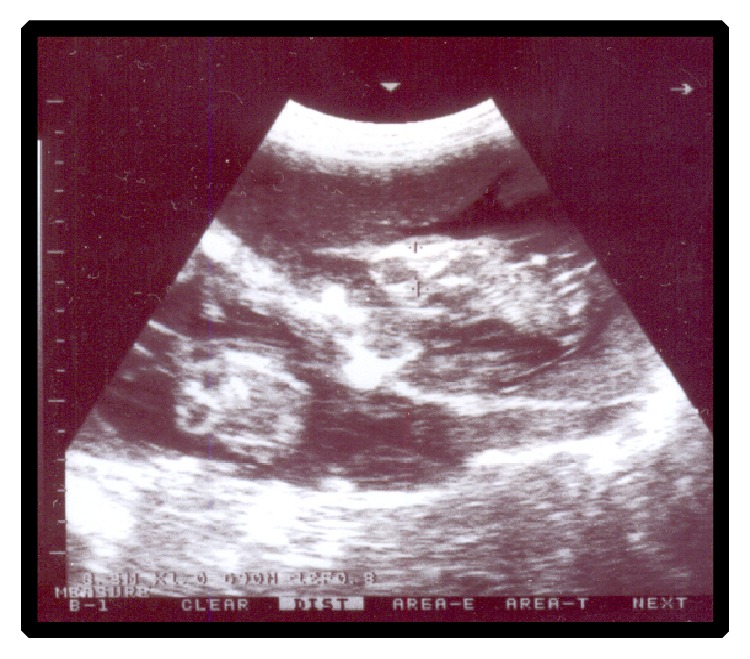
Dead twins at 60 days of gestation (arrows).

**Figure 2:**
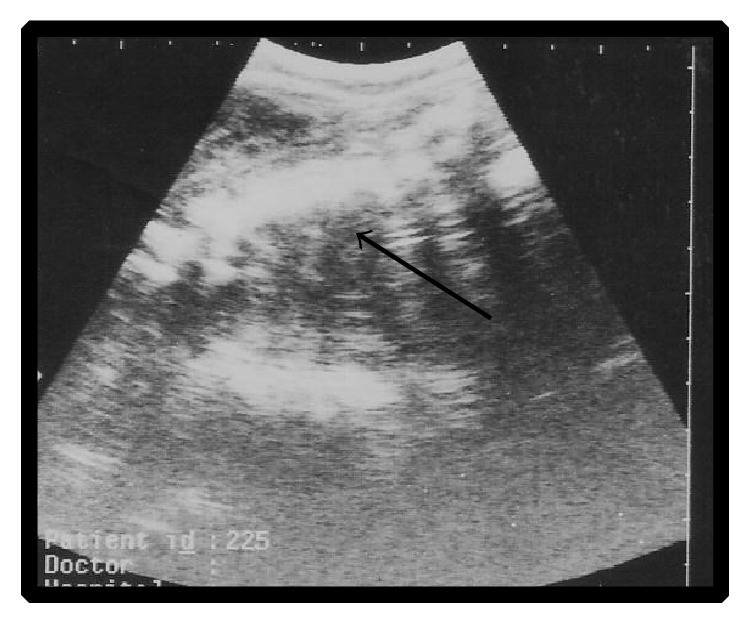
Single dead fetus at 120 days of gestation (arrow).

Ultrasonographic examination of the thirteen goats revealed non-viable fetuses characterized by absence of heart beats and fetal movements. Regarding the full-term goats with dead fetuses, fetal fluids were greatly diminished and distal acoustic shadowing was clearly realized. Out of thirteen goats, two were diagnosed as having dead twins (Figure 1) and 11 were diagnosed as having single dead fetus (Figure 2). Three goats were diagnosed at gestational age of about 60 days, three at about 40 days, four at about 120 days, and three goats at full term.

### 3.2. Treatment and Consequent Fertility

**Figure 3:**
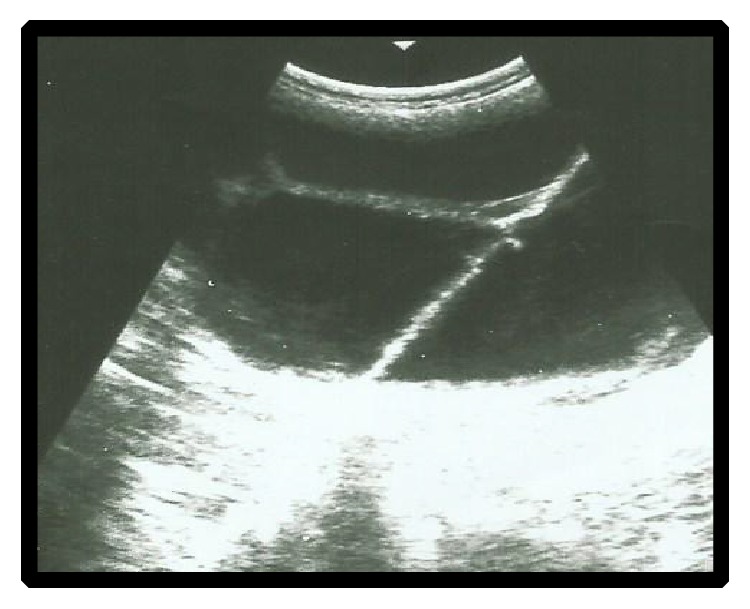
Hydrometra.

**Figure 4:**
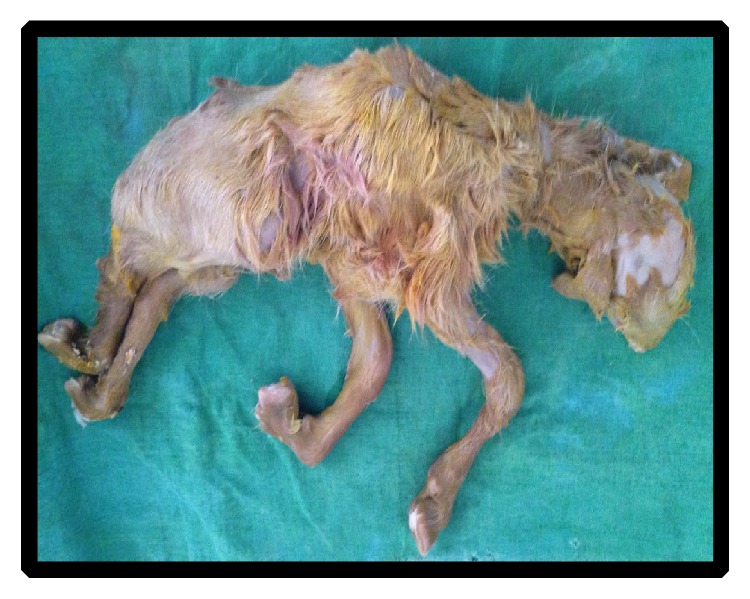
Full-term dead fetus removed by cesarean section.

Twelve goats were treated using intramuscular injection of PGF_2*α*_ analogue (Estrumate 125 *μ*g i/m, Schering-Plough Animals Health, Germany) and oxytetracycline (5%) with a dose rate of 0.5 mL and 5 mL (for five consecutive days), respectively. After treatment, ten goats responded successfully to the treatment as judged by dilatation of the cervix, abortion of fetuses after 48–96 hours. The animals returned to estrus and the animals' owners were advised to breed their goats at the next coming oestrus cycle. Six out of ten goats were diagnosed later as pregnant as confirmed by ultrasonography and gave birth at the normal gestational period. Two goats were not mated and thus the subsequent fertility was unknown. One goat was diagnosed as non-pregnant and one goat developed hydrometra (Figure 3). Two full-term goats did not respond to the treatment and another dose of PGF_2*α*_ was administered. The goats did not respond to the treatment for the second time and hence were referred to surgery. Regarding the third full-term goat with dilated cervix, more than a few attempts were made to deliver the goat, but all attempts were unsuccessful. Supportive treatment and broad-spectrum antibiotic injection was administered to the goat and also referred to surgery. Cesarean section and hysterectomy were performed for the three full-term goats and fetuses were removed (Figure 4) with unfavorable outcome.

## 4. Discussion

Pregnancy diagnosis can be performed accurately using real-time ultrasonography. Transabdominal ultrasound can detect the embryo with its heart beat by day 27 of gestation [26]. Fertilization rate in domestic animals is generally very high; however, as many as 65% are lost during embryonic and fetal development [19].

The most serious curtailment of efficient animal production and herd profitability is pregnancy loss [27]. Reproductive efficiency was affected by fertilization failure and embryonic mortality with the latter being the more significant [28]. Prenatal death is divided into embryonic and fetal death [3]. Embryonic mortality denotes the death of fertilized ova and embryos up to the end of implantation [29].

In early pregnancy stages, dying embryos usually undergo a partial degeneration process before being expelled [30]. Several factors have been implicated in embryo and fetal loss and are normally categorized as those of genetic, physiological, endocrine, and environmental origin [31]. Results of the current study revealed that death can occur at any time during gestation; however, the exact outcome of fetal mortality is unpredictable and influenced by several factors, such as the cause of the death, stage of gestational age, and number of fetuses [17]. Results showed that fetal death occurring during 2–4 months had excellent consequence; however, fetal death at full-term gestation had unfavorable outcome. It was concluded that real-time ultrasonography is a rapid, accurate, and nondisruptive method for diagnosis of antenatal death and prostaglandin F_2*α*_ and oxytetracycline proved to be efficient treatment for fetal death during 2–4 months of gestation.
